# Radius exponent in elastic and rigid arterial models optimized by the least energy principle

**DOI:** 10.1002/phy2.236

**Published:** 2014-02-17

**Authors:** Yoshihiro Nakamura, Shoichi Awa

**Affiliations:** 1 Department of Pediatrics, Graduate School of Medicine, University of Tokyo, 7‐3‐1 Hongo, Bunkyo‐ku, 113‐8655, Tokyo, Japan; 2 (Formerly) Department of Pediatrics, Kyorin University School of Medicine, 6‐20‐2 Shinkawa, Mitaka city, 181‐8611, Tokyo, Japan

**Keywords:** Bernoulli's equation, Murray's law, optimality principle, Poiseuille's law, Reynolds number

## Abstract

It was analyzed in normal physiological arteries whether the least energy principle would suffice to account for the radius exponent *x*. The mammalian arterial system was modeled as two types, the elastic or the rigid, to which Bernoulli's and Hagen‐Poiseuille's equations were applied, respectively. We minimized the total energy function *E*, which was defined as the sum of kinetic, pressure, metabolic and thermal energies, and loss of each per unit time in a single artery transporting viscous incompressible blood. Assuming a scaling exponent *α* between the vessel radius (*r*) and length (*l*) to be 1.0, *x* resulted in 2.33 in the elastic model. The rigid model provided a continuously changing *x* from 2.33 to 3.0, which corresponded to Uylings’ and Murray's theories, respectively, through a function combining Reynolds number with a proportional coefficient of the *l *− *r* relationship. These results were expanded to an asymmetric arterial fractal tree with the blood flow preservation rule. While *x* in the optimal elastic model accounted for around 2.3 in proximal systemic (*r *>**1 mm) and whole pulmonary arteries (*r *≥**0.004 mm), optimal *x* in the rigid model explained 2.7 in elastic‐muscular (0.1 < *r *≤**1 mm) and 3.0 in peripheral resistive systemic arteries (0.004 ≤ *r *≤**0.1 mm), in agreement with data obtained from angiographic, cast‐morphometric, and in vivo experimental studies in the literature. The least energy principle on the total energy basis provides an alternate concept of optimality relating to mammalian arterial fractal dimensions under *α *= 1.0.

## Abstract (precis)

The least energy principle explains the radius exponent x of each elastic and rigid arterial system. Assuming that a scaling exponent between *l *− *r* relationship is equal to 1.0, *x* in the optimal elastic arterial model resulted in 2.33. The optimal rigid arterial model provided a continuously changing *x* from 2.33 to 3.0, which corresponded to Uylings’ and Murray's theories, respectively, through a function combining Reynolds number with a proportional coefficient of the *l *− *r* relationship.

## Introduction

Mean aortic blood pressure falls only 2% from the ascending aorta to small arteries whose inner radius (*r*) narrows to ~1 mm (Struijker‐Boudier 2009) (or ~2.5 mm, Nichols et al. 2011) in the human systemic circulation (Nichols et al. 2011). The normal pulmonary arterial circulation shares 50% (Brody et al. 1968; Bhattacharya and Staub 1980; Michel et al. 1985) of the whole mean transpulmonary pressure gradient, which is as low as 6–7 mmHg in humans (Fowler 1980; Kovacs et al. 2009; Nakamura et al. 2011). The common factor in these two small pressure‐losing systems lies largely in the elasticity of its arterial wall (Patel et al. 1960; Learoyd 1966; Gow and Taylor 1968; Milnor 1982; Zhuang et al. 1983; Al‐Tinawi et al. 1991; Gan and Yen 1994; Dawson et al. 1999; Nichols et al. 2011). The sufficient elasticity and resultant large distensibility in proximal systemic arteries are due to the large ratio of constituent elastin over collagen and the thin smooth muscle layer (Struijker‐Boudier 2009; Nichols et al. 2011). On the other hand, the pulmonary arterial wall is much thinner, less‐or‐non muscularized, and more distensible than its systemic counterpart (Patel et al. 1960; Gow and Taylor 1968; Milnor 1982; Al‐Tinawi et al. 1991; Guyton 1991; Dawson et al. 1999; Nichols et al. 2011). Thus, even though peripheral pulmonary arteries of *r *≤**0.1 mm comprise the most resistive in the pulmonary circulation (Bhattacharya and Staub 1980; Michel et al. 1985), the mean pressure gradient through them amounts to only 2.8 mmHg, as estimated in controls of our clinical data (Nakamura et al. 2011).

By contrast, as much as 60% of mean aortic blood pressure is lost in peripheral systemic resistive arteries (0.004 ≤ *r *≤**0.1 mm) (Nichols et al. 2011). A much smaller elastin/collagen ratio in more peripheral systemic arteries renders their wall a lot stiffer (Learoyd 1966; Gow and Taylor 1968; Struijker‐Boudier 2009; Nichols et al. 2011). Furthermore, adding a thick smooth muscle layer to this already stiffer wall property makes the change in the radius in response to internal pulsatile pressure alteration extremely small in systemic arterioles (Meyer et al. 1988; Nichols et al. 2011).

Some histologists, however, advocate another intermediate category of systemic arteries around 0.1 < *r*_s_ ≤ 1 mm (Struijker‐Boudier 2009) (or ≤2.5 mm, Nichols et al. 2011), where the pressure gradient comprises 8% of mean aortic pressure in humans (Nichols et al. 2011). They describe it as elastic‐muscular and its wall property lies inbetween the more proximal elastic and more peripheral resistive arteries (Struijker‐Boudier 2009; Nichols et al. 2011).

In blood vessels where blood transportation is their main function, mean blood volume flow per unit time (*q*) through a cylindrical vessel has been empirically expressed as a function of its internal vessel radius *r*, as shown in equation 1 with a real number *x*:
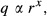
which is called the flow‐radius relationship (Mayrovitz and Roy 1983; Woldenberg 1983; House and Lipowsky 1987; Horsfield and Woldenberg 1989; Bennett et al. 2000). Hereafter, unless otherwise indicated, the internal radius is simply described as the radius, and all the variables and parameters are expressed in the International System of Units (SI) in equations. When a mother vessel branches into two daughter vessels, representing the blood flow of the mother and of the two daughters as *q*_m_, *q*_d1_, and *q*_d2_, respectively, the preservation of blood flow through these three vessels gives the following
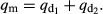


Moreover, indicating the radii of the mother and her two daughter vessels as *r*_m_, 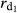, and 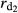, respectively, equations (1 and 2 provide us with the relationship between them as
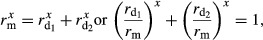
where *x* is called the radius exponent (Mandelbrot 1983; Kamiya and Takahashi 2007; Nakamura et al. 2011) or the junction exponent (LaBarbera 1995; Bennett et al. 2000), and corresponds to the fractal dimension of embedding in fractal theory for an asymmetric vascular tree applicable to multiple consecutive arterial generations (Suwa and Takahashi 1971; Mandelbrot 1983; West et al. 1997; Bennett et al. 2000; Gafiychuk and Lubashevsky 2001; Zamir 2001; Kamiya and Takahashi 2007; Nakamura et al. 2011). In normal physiological situations, the mean of *x* stayed between 2.0 and 3.0, although its range was reported to be from as low as 1.0 to over 4.0 through various methods in a variety of mammalian arteries (Suwa and Takahashi 1971; Sherman 1981; Mayrovitz and Roy 1983; Woldenberg 1983; House and Lipowsky 1987; Horsfield and Woldenberg 1989; Kassab and Fung 1995; LaBarbera 1995; Dawson et al. 1999; Bennett et al. 2000; Zamir 2001; Ghorishi et al. 2007; Nakamura et al. 2011). Hereafter we use suffixes s and p to indicate systemic and pulmonary, respectively.

**Figure 1.**
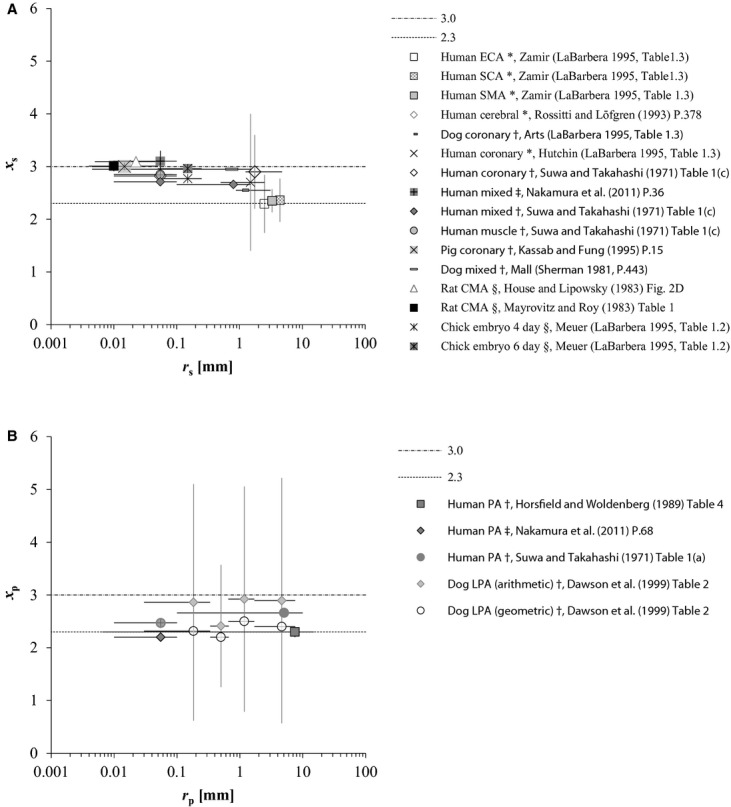
(A) Distribution of reported *x*_s_ in a variety of animals by previous studies through various methods, all of which, except for Meuer's data (LaBarbera 1995), are humans (Singhal et al. 1973; Rossitti and Löfgren 1993; LaBarbera 1995; Nakamura et al. 2011) and mammals (Sherman 1981; Mayrovitz and Roy 1983; House and Lipowsky 1987; Kassab and Fung 1995). CMA, ECA, SCA, and SMA indicate the cremaster muscle, external carotid, subclavian, and superior mesenteric arteries, respectively. Radii of ECA, SCA, and SMA were estimated from Olufsen et al.'s (2000) table 1 as 2.5, 4.4, and 3.3 mm, respectively; radius of ECA was tentatively substituted for the mean of the minimal radius at the outlet of bilateral common carotid arteries. House and Lipowsky's (1987) result presented in this figure was derived from the volumetric flow of red blood cells. (B) Distribution of reported *x*_p_ in humans (Suwa and Takahashi 1971; Horsfield and Woldenberg 1989; Nakamura et al. 2011) and dogs (Dawson et al. 1999). PA, pulmonary arterial tree; LPA, left PA. *r*, vessel radius, presented at the mid‐point with the range because the mean and median were not reported in the literature; *x*, radius exponent, defined by in equation (1 or 3 and presented as the mean with one standard deviation. Suffixes s and p indicate systemic and pulmonary. Methodology is indicated by symbols: *angiography; ^†^cast morphometry; &^ddagger;^model analysis with catheter data; ^§^direct measurement in vivo.

Previously reported results of *x*_s_ are plotted in Figure 1A against the corresponding *r*_s_, for which we could identify the range. These data mainly reflect *r*_s_ – *x*_s_ sets listed in LaBarbera's (1995) review article, and include those reported by other studies (Suwa and Takahashi 1971; Sherman 1981; Mayrovitz and Roy 1983; House and Lipowsky 1987; Rossitti and Löfgren 1993; Kassab and Fung 1995; Nakamura et al. 2011). *x*_s_ ≈ 2.0–2.3 was reported by angiographic morphometry in proximal systemic arterial branching structures, such as from the aorta to next‐generation large arteries (Zamir and Brown 1982; Zamir et al. 1992; LaBarbera 1995), where little pressure drop takes place. In contrast, *x*_s_ ≈ 3 has consistently been reported in peripheral systemic resistive arteries of 0.004 ≤ *r*_s_ ≤ 0.1 mm (Nichols et al. 2011) by postmortem cast morphometry in mammals (Sherman 1981; Kassab and Fung 1995) and direct measurement of the *q*_s_ − *r*_s_ relationship in in vivo rat cremaster arteries (Mayrovitz and Roy 1983; House and Lipowsky 1987). The result of our recent analysis using human hemodynamic data also gave *x*_s_ = 3.1 ± 0.2 in peripheral systemic resistive arterial trees, whose radius was assumed to range from 0.01 to 0.1 mm (Nakamura et al. 2011). However, transitional or intermediate values of *x*_s_ ≈ 2.7 have also been observed in several organs of some mammalian systemic arteries (Suwa and Takahashi 1971; LaBarbera 1995; Bennett et al. 2000).

**Figure 2.**
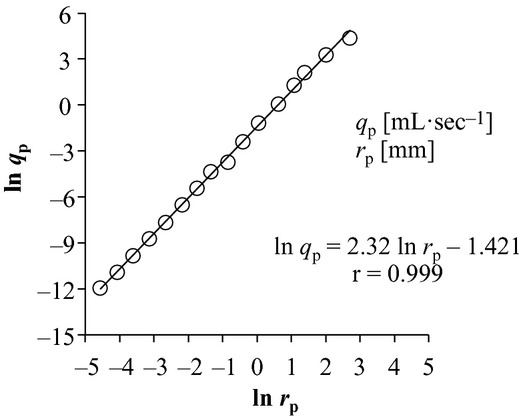
Ln‐ln plot of human pulmonary arterial blood flow (*q*_p_) in a vessel against its corresponding vessel radius (*r*_p_). The original data for the plot are derived from combining Singhal et al.'s (1973) tables 4 and 5 under the condition of assumed cardiac output of 4.8 L/min. The slope of this proportionality is equal to radius exponent *x*. r indicates the correlation coefficient.

By the same token, we present the pulmonary arterial counterpart reported in humans and dogs in the literature (Suwa and Takahashi 1971; Horsfield and Woldenberg 1989; Dawson et al. 1999; Nakamura et al. 2011) in Figure 1B. Mean *x*_p_ was reported to be 2.3 ± 0.1 (0.0065 ≤ *r*_p_ ≤ 15 mm) (Horsfield and Woldenberg 1989), or to range from 2.47 ± 0.09 (*r*_p_ < 0.1 mm) to 2.66 ± 0.07 (*r*_p_ ≥ 0.1 mm) by pulmonary arterial cast morphometry of normal humans (Suwa and Takahashi 1971). Assuming that the peripheral pulmonary arterial radius ranges from 0.01 to 0.1 mm in common with the systemic counterpart, our recent report indicated *x*_p_ = 2.2 in peripheral pulmonary arteries in normal humans (Nakamura et al. 2011). The relationship between *q*_p_ and *r*_p_ in the human pulmonary arterial tree simulated by Singhal et al. (1973) on the basis of their cast morphometry is also presented with a log–log plot in Figure 2, where the slope of this plot clearly indicates that *x*_p_ stays constant at 2.32 starting from proximal large to peripheral small arteries. However, a marked large standard deviation (SD) of the arithmetic mean of *x*_p_ around 2.9 was also reported in dog lungs by Dawson et al.'s (1999) cast‐morphometric study, as indicated in Figure 1B.

To date, the most influential and prevailing theory for *x*_s_ = 3 found in the peripheral systemic arterial bed (Uylings 1977; Sherman 1981; Zamir and Brown 1982; Rossitti and Löfgren 1993; Kassab and Fung 1995; LaBarbera 1995; Nakamura et al. 2011) is Murray's law, which applied the minimum cost principle to a rigid cylindrical artery with viscous Newtonian steady flow (Murray 1926; Uylings 1977; Sherman 1981; Mayrovitz and Roy 1983; Griffith and Edwards 1990; LaBarbera 1995; Gafiychuk and Lubashevsky 2001; Nakamura et al. 2011). Fractal space‐filling embedding (Mandelbrot 1983; Gafiychuk and Lubashevsky 2001) also provides an alternative theoretical basis for *x*_s_ = 3. However, these two principles are not by themselves effective enough to explain the consistency of the radius exponent of the proximal systemic (*r*_s_ > 1 [or 2.5] mm) (Zamir and Brown 1982; Zamir et al. 1992; LaBarbera 1995), intermediate systemic elastic‐muscular arteries (0.1 < *r*_s_ ≤ 1 [or 2.5] mm) (Suwa and Takahashi 1971; LaBarbera 1995), or the pulmonary arterial beds (Singhal et al. 1973; Horsfield and Woldenberg 1989; Nakamura et al. 2011) on the common basis. Several explanations for *x* from 2.0 to 2.7 have since been attempted, such as the cross‐sectional area‐preserving law (*x* = 2.0) (Woldenberg 1983; Zamir et al. 1992; LaBarbera 1995; West et al. 1997; Bennett et al. 2000), minimization of both drag and power loss (*x* = 2.0) (Griffith and Edwards 1990; Bennett et al. 2000), complete turbulence (*x = *7/3 = 2.33) (Uylings 1977; Bennett et al. 2000), minimization of surface area and power loss (*x = *2.5) (Griffith and Edwards 1990; Bennett et al. 2000), and minimum volume principle (2.1 < *x *<**2.8) (Woldenberg 1983; Bennett et al. 2000).

Hagen‐Poiseuille's equation has long been used universally to express the pressure gradient in arterial models including Murray's theory, irrespective of whether *r* is derived from big arteries like aorta or from small peripheral arterioles (Murray 1926; Suwa and Takahashi 1971; Uylings 1977; Sherman 1981; Mayrovitz and Roy 1983; Rossitti and Löfgren 1993; Kassab and Fung 1995; LaBarbera 1995; West et al. 1997; Dawson et al. 1999; Gafiychuk and Lubashevsky 2001; Kizilova 2006; Ghorishi et al. 2007; Kamiya and Takahashi 2007; Nakamura et al. 2011). However, it is also well known that Hagen‐Poiseuille's equation is unable to accurately estimate vascular resistance in proximal systemic elastic arteries and whole pulmonary arteries because of their large pulsatile fluctuation of radius (Horsfield and Woldenberg 1989; Middleman 1995; Nichols et al. 2011). On the other hand, Bernoulli's equation can and should rather reasonably be applied to blood flow through elastic arteries, such as proximal human systemic or whole pulmonary arteries (Lima et al. 1983a,b; Bermejo et al. 2002; Nichols et al. 2011).

The present article tries to introduce an alternate novel theory for optimal arterial models to explain various values of the radial exponent, which were observed in normal, namely, physiological mammalian arteries from the standpoint of the least energy principle. Prior to the theoretical analysis to follow, referring to Figure 1A and B, we tentatively reviewed the hitherto reported locality, wall property, and radius exponent of systemic and pulmonary arteries, which had been categorized by the radius alone, as shown in Table 1 A and B, because it was considered to provide an overview of the present status of the whole situation surrounding the radius exponent and related morphology.

**Table 1.** Categorization of mammalian arteries by locality in terms of radius, arterial wall property, and consequently radius exponent.

Locality	Range of radius (mm)	Wall property	*x*_s_	References
(A) *Systemic*				
Proximal	1 < *r*_s_	Elastic^1^	~2.3	Zamir and Brown (1982); Zamir et al. (1992); LaBarbera (1995)
Intermediate	0.1 < *r*_s_**≤ 1	Elastic‐muscular^2^	~2.7	Suwa and Takahashi (1971); LaBarbera (1995)
Peripheral	0.004 ≤ *r*_s_ ≤ 0.1	Muscular (rigid)^2^	~3.0	Sherman (1981); Mayrovitz and Roy (1983); House and Lipowsky (1987); Kassab and Fung (1995); Nakamura et al. (2011)

Locality	Range of radius (mm)	Wall property	*x*_p_	References
(B) *Pulmonary*				
Proximal	0.1 < *r*_p_	Elastic^1^	~2.3	Singhal et al. (1973); Horsfield and Woldenberg (1989); Dawson et al. (1999)
Peripheral	0.004 ≤ *r*_p_ ≤ 0.1	Elastic^1^	~2.3	Singhal et al. (1973); Horsfield and Woldenberg (1989); Dawson et al. (1999); Nakamura et al. (2011)

The range of the radius presented in each classification of systemic and pulmonary arteries comes from Struijker‐Boudier's (2009) figure 1 and Suwa and Takahashi's (1971) table 1(a), respectively. *r* stands for radius; *x*, radius exponent defined by equation (1 or 3. Suffixes p and s indicate pulmonary and systemic, respectively. Although the lower limit of the vessel radius is common to that of capillaries, our arterial model does not conceptually include the capillary vessel.

^1,2^indicate vessel types to which we applied our elastic and rigid arterial models, respectively. Data of *x* came from table 1 (Dawson et al. 1999), table 4 (Horsfield and Woldenberg 1989), figure 2D (House and Lipowsky 1987), P. 15 (Kassab and Fung 1995), table 1.3 (LaBarbera 1995), table 1 (Mayrovitz and Roy 1983), P. 36 (Nakamura et al. 2011), P. 443 (Sherman 1981), tables 4 and 5 (Singhal et al. 1973), and table 3 (Suwa and Takahashi 1971).

First, we theoretically used Murray's theory by approaching through the *least energy principle*, where Bernoulli's and Hagen‐Poiseuille's equations were, respectively, applied in elastic and rigid arterial models, and we tried to delineate the respective optimal arterial design of these two models in the normal physiological circulation. Second, we also estimated *x*_s_ and *x*_p_ through mathematical and fractal analysis using previously reported morphometric data of systemic as well as pulmonary arterial trees in the literature. Finally, theoretical results were compared with either reported and/or our own estimated results and discussed in relation to conventional theories.

## Methods

### Theoretical background

Murray (1926) deliberated on the cost function *C* for a steady blood flow in a cylindrical artery and came up with the sum of pressure energy loss (Δ*U*_P_) per unit time, as defined by Hagen‐Poiseuille's equation on one hand, and the loss of metabolic energy (Δ*U*_M_) of blood in the vessel per unit time on the other. Indicating pressure loss through the vessel, blood flow rate, internal vessel radius, vessel length, and blood viscosity as Δ*P*,* q*,* r*,* l*, and *μ*, respectively, Δ*P* and Δ*U*_P_ were represented as
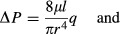

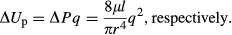


To extend Δ*U*_M_ from the original definition by Murray (1926), which was intended only for blood volume in a vessel, to the whole vascular volume, Mayrovitz and Roy (1983) later added the metabolic term of an arterial wall (the second term in the next eq. 6 to that of blood only (the first term) for Δ*U*_M_. Defining the metabolic rate of blood per unit time and volume, that of the vessel wall, and the wall thickness of blood vessels as *K*_b_, *K*_w_, and *h* (Mayrovitz and Roy 1983), respectively, Δ*U*_M_ was represented anew as
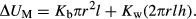


The wall thickness *h* can be assumed to be constantly proportional to *r* from large arteries down to micro arterioles and given by *h *= *wr*, where *w* is reported by past morphometric studies to be 0.25 in systemic large arteries and 0.2–0.5 in small arteries and arterioles (Uylings 1977; Mayrovitz and Roy 1983; Nichols et al. 2011). The wall thickness of pulmonary arteries is estimated as one‐third of their systemic counterparts (Guyton 1991). Hence, equation 6 can be rewritten with the aggregated metabolic rate *K* as
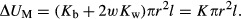


Combining equations (5 and 7, Murray's cost function *C* was defined as (Murray 1926; Mayrovitz and Roy 1983)
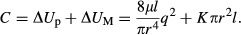


Applying partial differentiation of *C* to equation 8 with respect to *r*, the condition of ∂*C/*∂*r* = 0 on the basis of *minimum cost principle* leads to 16*μq*^2^/(*Kπ*^2^) = *r*^6^, which then results in the next equation 9:
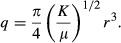


This cubic relationship between *q* and *r* is Murray's law itself. However, this persuasive explanation does not necessarily guarantee the minimum cost of the whole arterial tree, where arterial ramifications are essential.

### Definition of energy function for an artery

We designed the energy function for a single artery and designated it *E*, which represents the whole energy needed to drive and transport blood, and maintains both the vessel and the blood. Blood flow was treated as a viscous incompressible Newtonian fluid. Time average or the mean of kinetic, pressure, and metabolic and thermal energies of blood flow are defined as *U*_K_, *U*_P_, and *U*_M_, respectively; the arterial system transports these three main energies along the stream, as will be detailed later. *U*_M_ does not include the metabolic and thermal energy held by the arterial wall in this model, because it is not transported by the arterial stream. Mean losses of kinetic, pressure, and metabolic and thermal energies through the vessel per unit time are represented as Δ*U*_K_, Δ*U*_P_, and Δ*U*_M_, respectively, while Δ*U*_K_ and Δ*U*_P_ are not significantly dependent upon the shape of branching in this model. We assume that there is no interconversion between dynamic energy (*U*_K_, *U*_P_, Δ*U*_K_, and Δ*U*_P_) and metabolic and thermal energy (*U*_M_ and Δ*U*_M_) within the vessel. Energy dissipation due to turbulence of blood flow is not taken into consideration either in this model. The potential energy held by the gravitational center of blood flow and its change through a single artery are not treated separately in this model. While the metabolic energy is partly converted to heat in the whole vascular volume including blood, the movement of heat is assumed to be in equilibrium between the arterial system and the surrounding tissue. The energy conservation through this single artery is expressed as
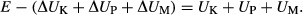
Thus, we get
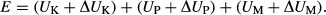


Therefore, *E* is defined as the sum of kinetic energy, pressure energy, metabolic and thermal energy, and loss of each. The rigorous cost function *C* is definable in equation 11 as
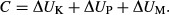


Minimizing *E* optimizes the arterial design of this model analysis. However, neither this optimization nor Murray's model incorporates and so guarantees the minimum cost at the arterial branching.

### Energy function for an elastic artery

The elastic arterial model is assumed to represent both proximal systemic arteries (*r*_s_ > 1 mm) (Struijker‐Boudier 2009) and the whole pulmonary arterial tree spanning from proximal (*r*_p_ > 0.1 mm) to peripheral (0.004 ≤ *r*_p_≤ 0.1 mm) (Singhal et al. 1973; Horsfield 1978; Horsfield and Woldenberg 1989; Huang et al. 1996; Dawson et al. 1999), as indicated also in Table 1A and B. The elastic arterial model is basically assumed cylindrical but might have a modicum of tapering toward the end (Milnor 1982; Dawson et al. 1999; Nichols et al. 2011).

The means of the internal vessel radius and length over time and space in a single elastic artery are indicated as *r* and *l*, and the mean blood volume flow per unit time and the mean pressure over time and space are also represented as *q* and *P*, respectively. The pressure drop produced along the length of the vessel is expressed by Δ*P*.

Indicating the specific gravity of blood as *ρ*, the mean mass of blood volume flow per unit time is given by *ρq*. The mean linear velocity *v* at the gravitational center of blood flow is given as *q*/*πr*^2^. Thus, *U*_K_, the kinetic energy of blood flow through the vessel per unit time is expressed in the next equation 13 (Milnor 1982):
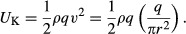


In an elastic artery with a modicum of tapering, Bernoulli's effect provides an increase in *U*_K_, which makes Δ*U*_K_ negative. *U*_P_ and Δ*U*_P_ are described as shown below (Milnor 1982):
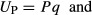

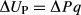


Because 1 *μ*L O_2_ consumption corresponds to 5 × 10^−3^ cal (=2.1 × 10^−2^ J) of metabolic energy (Mayrovitz and Roy 1983), *U*_M_ is defined as the sum of the metabolic energy converted from the oxygen supply of arterial blood flow and genuine thermal energy in it:
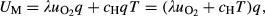
where *λ*, 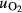, *c*_H_ and *T* indicate the proportional coefficient to convert oxygen volume in blood to equivalent metabolic energy (*λ* = 2.1 × 10^4^ J L^−1^), the arterial oxygen volume per unit blood volume, the specific heat capacity of blood per unit volume, and the mean absolute temperature of blood as an average over time and space, respectively. 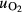 is regarded as constant throughout the arterial tree in this model for simplification.

Furthermore, Δ*U*_M_ in this article is defined again in equation 7 as in Murray's or Mayrovitz and Roy's equations; however, the *l *− *r* relationship of an arterial tree in a number of human organs has already been reported by Suwa and Takahashi (1971), who indicated that *l* was a function of *r* using real numbers *α* and Ω in both systemic and pulmonary arteries as
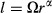


This empirical expression has been applied widely in arterial fractal models (Suwa and Takahashi 1971; West et al. 1997; Dawson et al. 1999; Olufsen et al. 2000; Gafiychuk and Lubashevsky 2001; Kizilova 2006; Kamiya and Takahashi 2007). The morphologically estimated value of the exponent *α* centers around 1.0, ranging from 0.76 to 1.21, in various human systemic (Suwa and Takahashi 1971; Kamiya and Takahashi 2007) as well as mammalian pulmonary arteries (Suwa and Takahashi 1971; Dawson et al. 1999), as listed in Table 2. Simply assumed to be 1.0, it has generally been used and discussed in model studies (Suwa and Takahashi 1971; West et al. 1997; Dawson et al. 1999; Olufsen et al. 2000; Gafiychuk and Lubashevsky 2001; Kizilova 2006; Kamiya and Takahashi 2007). Therefore, when we need to deal with *α* in this model analysis, *α* is set tentatively to 1.0 for the sake of simplicity and brevity. As a result, equation 7 was rewritten with equation 17 as
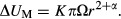
Substitution of equations (13–16 and 18 into equation 11 gives
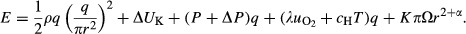


**Table 2.** Reported data of *α* and Ω in systemic and pulmonary arteries by previous studies of fractal analysis with cast‐morphometric measurements in the literature.

	Reference	Range of radius (mm)	*α*	Ω
Systemic				
Human renal	Suwa and Takahashi (1971)	≥0.01	0.85	17.6
Mesenteric	Suwa and Takahashi (1971)	≥0.01	1.04	13.0
Femoral	Suwa and Takahashi (1971)	≥0.01	1.01	13.2
Pancreas	Suwa and Takahashi (1971)	≥0.01	0.90	16.1
Cerebral cortex	Suwa and Takahashi (1971)	≥0.01	1.15	7.4
Basal ganglion	Suwa and Takahashi (1971)	≥0.01	1.21	4.6
Coronary	Suwa and Takahashi (1971)	≥0.01	1.05	7.9
Pulmonary				
Human^1^	Dawson et al. (1999)	0.0065–0.425	0.85	6.43
Human^2^	Dawson et al. (1999)	0.01–7.4	0.89	9.51
Human	Suwa and Takahashi (1971)	≥0.01	1.16	2.8
Dog lt.	Dawson et al. (1999)^5^	0.030–7.574	1.139–1.15	3.987–5.0
Dog	Dawson et al. (1999)		1.00	
Dog^3^	Dawson et al. (1999)	0.014–5.56	0.84	9.72
Cat	Dawson et al. (1999)		1.03	15.5
Rat^4^	Dawson et al. (1999)	0.00665–0.8	1.03	5.3

*α* and Ω represent exponent and proportional coefficient of the relationship between vessel length and radius, respectively, defined by equation (16); lt, left. Research articles which Dawson et al. (1999) used for their estimation are partly common to our references (Horsfield 1978; Gan and Yen 1994; Jiang et al. 1994; Huang et al. 1996).

^1,2,3,4^corresponded to the above‐mentioned Horsfield (1978); Huang et al. (1996); Gan and Yen (1994); Jiang et al. (1994), respectively. Sources of data were table 3 (Suwa and Takahashi 1971) and table 1 (Dawson et al. 1999), while ^5^means figure 4 (Dawson et al. 1999).

Because Bernoulli's effect always guarantees reciprocal conversion between Δ*U*_K_ and Δ*U*_P_ as Δ*U*_K_ + Δ*U*_P_ ≈ 0 throughout an elastic artery (Lima et al. 1983a,b; Bermejo et al. 2002; Nichols et al. 2011), Δ*U*_K_ + Δ*Pq* ≈ 0 also holds true in equation 19.

In conclusion, equation 19 is rewritten with Bernoulli's effect as
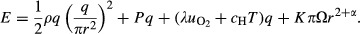


We partially differentiated *E* with respect to *r*, and applied ∂*E*/∂*r *=**0 to equation 20.

### Energy function for a rigid artery

The rigid arterial model is applied to intermediate (0.1 < *r*_s_ ≤ 1 mm) (Struijker‐Boudier 2009; Nichols et al. 2011) and peripheral systemic arteries (0.004 ≤ *r*_s_≤ 0.1 mm) (Mayrovitz and Roy 1983; House and Lipowsky 1987; Nichols et al. 2011), both of which we regard as cylindrical (Murray 1926; Suwa and Takahashi 1971; Uylings 1977; Sherman 1981; Mayrovitz and Roy 1983; Kassab and Fung 1995; LaBarbera 1995; West et al. 1997; Olufsen et al. 2000; Gafiychuk and Lubashevsky 2001; Kizilova 2006; Kamiya and Takahashi 2007; Nakamura et al. 2011; Nichols et al. 2011), as indicated in Table 1A. The energy function *E* in equation 11 was also applied to the analysis of the optimal design of a rigid artery. *U*_K_, *U*_P_, *U*_M_, and Δ*U*_M_ are similarly expressed as counterparts in equations (13, 14, 16, and 18, respectively. As Δ*U*_P_ in a rigid artery is due to the friction between viscous blood flow and the arterial inner surface (Nichols et al. 2011), Hagen‐Poiseuille's equation was applied as presented in equation 5. Because there is no change in mean linear velocity through a rigid artery due to Hagen‐Poiseuille's law, Δ*U*_K_ is equal to 0 in equation 11. By substituting equation 17 into equation 5 as Δ*U*_P_, *E* of a rigid cylindrical artery is finalized as 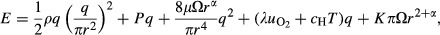
which involves the addition of Hagen‐Poiseuille's term in equations 5–9,5–20 as a consequence. Similarly, the optimal relationship between *q* and *r* was sought by ∂*E*/∂*r *=**0.

### Resources of subjected data

For comparison, we also analyzed mammalian arterial cast‐morphometric data sets reported in the literature, which contain *r*,* l*, and the number of vessels in an arterial tree in each generation. The rule of vessel generation is ranked and numbered at each ramification from the most proximal to the most peripheral arteries. However, if the literature in question happened to follow the rule of “order,” which adopted an inverse numbering system starting from the most peripheral arteries, we regarded the largest order as the first generation, revising orders into generations. One data set of canine mixed systemic arterial trees was available from Milnor's (1982) morphometric study. We also used Mall's data of canine superior mesenteric arterial tree modified by Sherman (1981), who adopted the rule of “rank,” which numbered the first‐generation vessel as 0, although Mall's data set lacks data about *l*. We could also acquire three human pulmonary arterial data sets from Horsfield's (1978), Huang et al.'s (1996), and Singhal et al.'s (1973) postmortem cast‐morphometric studies. Data in Huang et al. (1996) and Singhal et al. (1973) were from a 44‐year‐old man and a 32‐year‐old woman, respectively. Because table 6 in Horsfield (1978) was a revised data set of the human pulmonary arterial tree from table 4 of Singhal et al. (1973) by amending vessel numbers in small arteries (0.013 ≤ *r*_p_≤ 0.850 mm) (Horsfield 1978), our analysis did not include the data from order 11 to 17 (1.33 ≤ *r*_p_ ≤ 30 mm) in Horsfield (1978), which were common to those in Singhal et al. (1973). In addition, four mammalian data sets of the pulmonary arterial tree were subjected to our analysis; Gan and Yen's (1994) and Milnor's (1982) dog, Zhuang et al.'s (1983) cat, and Jiang et al.'s (1994) rat cast‐morphometric data. Data from two other studies (Olufsen et al. 2000; Nakamura et al. 2011) were also used.

### Data analysis of *x*,* α*, and Ω

Radial exponent *x* was estimated in each succeeding generation through the arterial tree of morphometric data sets. Letting *n* be a natural number, we assume that the *n*th generation mother arteries with mean internal radius *r*_*n*_ and mean vessel length *l*_*n*_ branch into the (*n *+**1)th generation daughter arteries with mean internal radius *r*_*n*+1_ and mean vessel length *l*_*n*+1_, while the vessel numbers of the *n*th and the (*n *+**1)th generation arteries are represented as *N*_*n*_ and *N*_*n*+1_, respectively. Defining the ratios of the radius, length, and vessel number of the (*n *+**1)th generation to the *n*th as *β*_*n*_ (0 <* β*_*n*_ < 1), *γ*_*n*_ (0 < *γ*_*n*_**< 1), and *η*_*n*_ (>1), we indicate the means of *β*_*n*_, *γ*_*n*_, and *η*_*n*_ through consecutive generations as *β*,* γ*, and *η*, respectively. Branching rules (West et al. 1997; Ghorishi et al. 2007; Nakamura et al. 2011) are given as
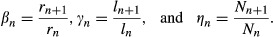


Using *x*_*n*_ as the radial exponent between *n*th and (*n *+**1)th generations, the preservation of blood flow through ramifications ensures the following
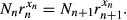


Equation 23 gives *x*_*n*_ as
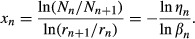


Means and SD of *x*_*n*_ were computed in respective arterial categories by the radius, as given in Table 1A and B, and are presented as estimated *x*.

*α* was estimated from equation 17 as the slope of linear regression analysis between ln*r* versus ln*l* (Suwa and Takahashi 1971; Dawson et al. 1999), where the vertical intercept corresponded to ln Ω throughout each arterial category as listed in Table 1A and B. By combining equations (17 and 22 as
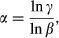


we alternatively sought *α* from the results of *β* and *γ*, which were reported in our previous study (Nakamura et al. 2011). We also computed Ω from Olufsen et al.'s (2000) data under *α *= 1.0.

### Statistical analysis

The decision of a statistically significant outlier was made by Dixon's Q test, which defines the estimator *Q* as a statistical value obtained by dividing the gap by the full range of sample data, where gap means the absolute difference between the outlier in question and the closest value to it (Böhrer 2008). When *Q* exceeded the critical value of confidential limit under *P* < 0.05, the outlier was excluded from further statistical analysis (Böhrer 2008).

## Results

### The elastic arterial system

Because ∂*E*/∂*r *=**0 in equation 20 results in 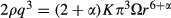, it follows
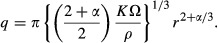


Hence, *x* was directly derived from equations (1 and 26 as follows:
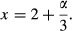


When *α* is assumed equal to 1.0, *x* is deduced as 2.33. Equation 3 is also written with equation 27 as
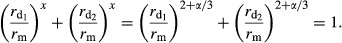


Using *α*,* β*,* γ*, and *η* on the basis of equations (22, 23, and 25, the preservation of blood flow extends equation 28 into an asymmetric fractal expression (Mandelbrot 1983; Kamiya and Takahashi 2007; Nakamura et al. 2011):
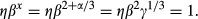


Equation 29 shows that *x* = 2 + *α*/3 of equation 27 retains its validity both structurally and functionally even in the case of more complex asymmetric ramifications.

### The rigid arterial system

∂*E*/∂*r *=**0 as applied to equation 21 gave
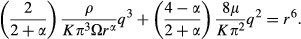


Letting *q*/*r* be *f* for convenience, *x* is given below in equation 31 by taking *α* as 1.0 only at the exponent in equation 30 (Appendix A1:
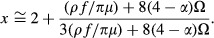


Using *v *= *q*/*πr*^2^, *f *= *q*/*r*, and *D* = 2*r*, where *D* stands for the internal vessel diameter, Reynolds number (*Re*) is defined and rewritten as shown in next equation 32 (Horsfield and Woldenberg 1989; Nichols et al. 2011):
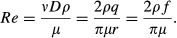


Eliminating *f* in equation 31 using equation 32, we can express *x* with *Re* and Ω as
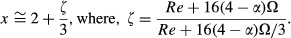


Equation 33 indicates that mean *x* asymptotically approaches its upper limiting value of 3.00 at *Re *< 10 and to its lower limiting value 7/3 = 2.33 at *Re* > 10^4^. Using *α*,* β*,* γ*, and *η* again, the blood flow preservation also guarantees equation 33 in an asymmetric arterial fractal by the same token as previously applied to the elastic arterial system:
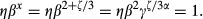


### Estimatin of *x*,* α*, and Ω from the literature

**Figure 3.**
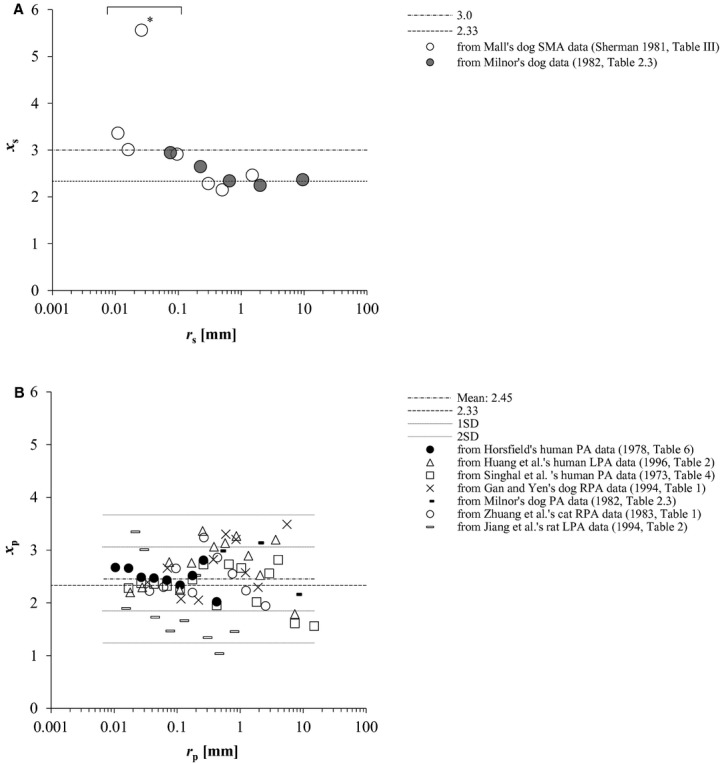
(A) Plot of estimated *x*_s_ vs. *r*_s_ at each arterial generation of systemic arteries of two dogs in the literature (Sherman 1981; Milnor 1982). *indicates the result regarded as an outlier by Q test (*P* < 0.05) (Böhrer 2008) among the estimates from Mall's data of dog superior mesenteric arteries (SMA) (Sherman 1981) at peripheral systemic arterial area (0.004 ≤ *r*_s_ ≤ 0.1 mm). (B) Counterparts of pulmonary arteries from three human (Singhal et al. 1973; Horsfield 1978; Huang et al. 1996) and four mammalian data (Milnor 1982; Zhuang et al. 1983; Gan and Yen 1994; Jiang et al. 1994) in the literature. Mean of all estimated *x*_p_ was indicated together with single and double standard deviations by broken and dotted lines, respectively. PA, pulmonary arterial tree; LPA, left PA; RPA, right PA. *r*, vessel radius; *x*, radius exponent, defined by in equation (1 or 3; s, systemic; p, pulmonary.

Distribution diagrams of the estimated *x*_s_ and *x*_p_ against *r*_s_ and *r*_p_, respectively, are presented in Figure 3A and B. Mean and SD of the estimated *x*_s_ and *x*_p_ in each categorized group by radius, as indicated in Table 1A and B are shown in Table 3A and B, respectively. Table 3A does not include the result of *x*_s_ = 5.56 at *r*_s_ = 0.026 mm from Sherman's rank 4 arteries in Mall's data (Sherman 1981) (our fifth generation equivalents) because the *Q* value resulted in 0.831 (= (5.56 − 3.36)/(5.56 − 2.91)) over the critical value of 0.829 (*P* < 0.05) (Böhrer 2008) among four estimates in the peripheral arterial area, as indicated in Figure 3A. Estimated *α* and Ω of systemic and pulmonary arterial trees are presented in Table 4A and B, respectively. Available systemic arterial data for this analysis were too scarce to separately estimate the parameters from these three groups, as classified in Table 1A.

**Table 3.** Estimated data of *x* in systemic (A) and pulmonary arteries (B) from reported data sets by previous studies.

	References	Range of *r*_s_ (mm)	*x*_s_
(A) *Systemic arteries*			
Proximal		1 < *r*_s_	
Dog	Milnor (1982)	2–9.5	2.32 ± 0.06
Dog SMA	Sherman (1981)	1.5	2.46
Intermediate		0.1 < *r*_s_**≤ 1	
Dog	Milnor (1982)	0.225–0.65	2.49
Dog SMA	Sherman (1981)	0.3–0.5	2.22 ± 0.09
Peripheral		0.004 ≤ *r*_s_**≤ 0.1	
Dog	Milnor (1982)	0.075	2.95
Dog SMA	Sherman (1981)	0.011–0.016	3.10 ± 0.24^1^

	References	Range of *r*_p_ (mm)	*x*_p_
(B) *Pulmonary arteries*			
Proximal		0.1 < *r*_p_	
Human	Horsfield (1978)	0.112–0.425	2.42 ± 0.33
Human lt.	Huang et al. (1996)	0.11–7.4	2.82 ± 0.50
Human	Singhal et al. (1973)	0.112–15	2.30 ± 0.46
Dog rt.	Gan and Yen (1994)	0.14–5.56	2.72 ± 0.57
Dog	Milnor (1982)	0.5–8.0	2.76 ± 0.52
Cat rt.	Zhuang et al. (1983)	0.176–2.54	2.50 ± 0.51
Rat lt.	Jiang et al. (1994)	0.13–0.8	1.60 ± 0.56
Peripheral		0.004 ≤ *r*_p_ ≤ 0.1	
Human	Horsfield (1978)	0.0065–0.069	2.54 ± 0.11
Human lt.	Huang et al. (1996)	0.01–0.075	2.42 ± 0.31
Human	Singhal et al. (1973)	0.011–0.069	2.33 ± 0.04
Dog rt.	Gan and Yen (1994)	0.014–0.070	2.52 ± 0.19
Cat rt.	Zhuang et al. (1983)	0.012–0.096	2.39 ± 0.23
Rat lt.	Jiang et al. (1994)	0.00665–0.078	2.29 ± 0.83

*r* stands for vessel radius; *x*, radius exponent, defined by equation (1 or 3; s, systemic; p, pulmonary. SMA indicates superior mesenteric artery; lt, left; rt., right. ^1^excluded the rank 4 arterial data (Sherman 1981) as an outlier. Vessels are categorized in terms of the radius by the classification presented in Table 1A and 1B. Data of the whole pulmonary arterial tree are indicated with no notation of laterality. The present analysis used table 1 (Gan and Yen 1994), table 6 (Horsfield 1978), table 2 (Huang et al. 1996), table 2 (Jiang et al. 1994), table 2.3 (Milnor 1982), table III (Sherman 1981), table 4 (Singhal et al. 1973), and table 1 (Zhuang et al. 1983). Data are presented as the mean with one standard deviation.

**Table 4.** Estimated data of *α* and Ω in systemic (A) and pulmonary arteries (B) by fractal analysis with published data in the literature.

	References	Range of radius (mm)	*α*_s_	Ω_s_	r
(A) *Systemic arteries*					
Human Ao	Olufsen et al. (2000)	7.2–12.0		3.3 ± 2.5^1^	
CCA		2.8–2.9		63.1 ± 5.0^1^	
Maj. brs.		1.9–7.0		17.9 ± 7.6^1^	
Human mixed	Nakamura et al. (2011)	0.01–0.1	0.78^2^		
Dog mixed	Milnor (1982)	0.025–9.5	1.11	70.66	0.99

	References	Whole tree			Proximal (0.1 mm < *r*_p_)			Peripheral (0.004 ≤ *r*_p_**≤ 0.1 mm)		
		*α*_p_	Ω_p_	r	*α*_p_	Ω_p_	r	*α*_p_	Ω_p_	r
(B) *Pulmonary arteries*										
Human	Horsfield (1978)	0.85	9.21	0.99	0.97	11.53	0.97	0.83	8.61	0.99
Human lt.	Huang et al. (1996)	0.89	9.38	0.98	0.86	9.69	0.96	0.55	2.61	0.98
Human	Nakamura et al. (2011)							0.71^2^		
Human	Singhal et al. (1973)	0.80	8.09	0.99	0.76	8.31	0.97	0.83	8.61	0.99
Dog rt.	Gan and Yen (1994)	0.83	13.13	0.99	0.77	13.30	0.97	0.92	16.51	0.99
Dog	Milnor (1982)	0.68	8.46	0.91						
Cat rt.	Zhuang et al. (1983)	1.03	14.11	0.99	0.78	13.01	0.95	1.21	24.77	0.99
Rat lt.	Jiang et al. (1994)	0.72	2.17	0.98	0.83	2.29	0.97	0.93	5.20	0.98

*α* and Ω represent exponent and proportional coefficients of the relationship between vessel length (*l*) and radius (*r*), respectively, defined by equation 17; r, correlation coefficient; s, systemic; p, pulmonary; lt., left; rt., right. Pulmonary arteries were categorized by radius; each range of the radius in subjected data is not indicated but is common to Table 3B. The data included in the present analysis came from table 1 (Gan and Yen 1994), table 6 (Horsfield 1978), table 2 (Huang et al. 1996), table 2 (Jiang et al. 1994), table 2.3 (Milnor 1982), P. 68 (Nakamura et al. 2011), table 1 (Olufsen et al. 2000), table 4 (Singhal et al. 1973), and table 1 (Zhuang et al. 1983). All methods but two (Olufsen et al. 2000; Nakamura et al. 2011) were cast‐morphometry; that of Nakamura et al. (2011), the combination of fractal‐model analysis and catheterization; that of Olufsen et al. (2000), measurement on magnetic resonance images or estimation. Ao stands for aorta, which includes ascending aorta, aortic arch, and abdominal aorta (Olufsen et al. 2000); CCA, common carotid arteries; Maj. brs., major branches, which include superior and inferior mesenteric, renal, internal and external iliac, and superficial and deep femoral arteries (Olufsen et al. 2000). Data of the whole pulmonary arterial tree are indicated with no notation of laterality.

^1^indicates estimates under *α *= 1.0.

^2^was calculated by equation 25. Data are presented as the mean with one standard deviation.

### Comparison of theoretically derived *x* with morphometrically determined *x*_*s*_ and *x*_p_

**Figure 4.**
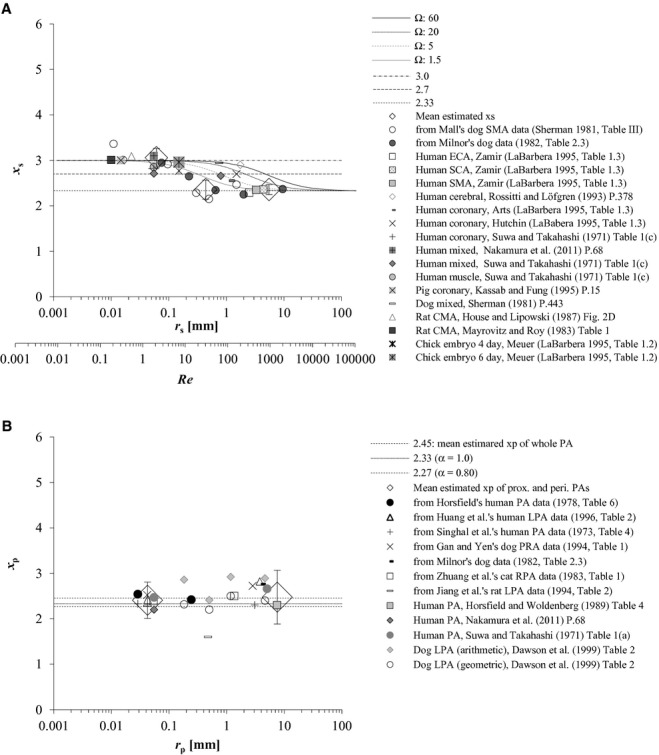
(A) Comparison between theoretical *x* from our rigid arterial model and morphometric *x*_s_. The model‐derived optimal relationship between the radius exponent *x* vs. Reynolds number (*Re*) in a rigid cylindrical artery was also plotted as curves based upon equation 33, which shift to the right with the increment of Ω. Ω represents the proportional coefficient of the vessel length‐radius relationship in equation 17. Both reported mean *x*_s_ in the literature (Suwa and Takahashi 1971; Sherman 1981; Mayrovitz and Roy 1983; House and Lipowsky 1987; Rossitti and Löfgren 1993; Kassab and Fung 1995; LaBarbera 1995; Nakamura et al. 2011) and those estimated from two dog data in previous studies (Sherman 1981; Milnor 1982) were plotted together against corresponding *r*_s_. Mean and one standard deviation (SD) of the estimates were plotted with large rhombuses and outliers, respectively, at proximal (*x*_s_, 2.36 ± 0.11; *r*_s_, 5.5 mm, range 1.5–9.5 mm), intermediate (*x*_s_, 2.36 ± 0.21; *r*_s_, 0.44 mm, range 0.23–0.65 mm), and peripheral arterial regions (*x*_s_, 3.06 ± 0.21; *r*_s_, 0.06 mm, range 0.01–0.10 mm), where Mall's rank 4 (corresponding to our 5th generation) arterial data (Sherman 1981) were excluded as an outlier. CMA, ECA, SCA, and SMA indicate the cremaster muscle, external carotid, subclavian, and superior mesenteric arteries, respectively. (B) Comparison between the elastic arterial model‐derived *x* and morphometric *x*_p_. Means of both reported *x*_p_ in the literature (Suwa and Takahashi 1971; Horsfield and Woldenberg 1989; Dawson et al. 1999; Nakamura et al. 2011) and those estimated from published data sets (Singhal et al. 1973; Horsfield 1978; Milnor 1982; Zhuang et al. 1983; Gan and Yen 1994; Jiang et al. 1994; Huang et al. 1996) were similarly plotted together against *r*_p_. Large rhombuses with outliers indicate the mean of estimated *x*_p_ with 1 SD at proximal (*x*_p_, 2.48 ± 0.59; *r*_p_, 0.67 mm, range 0.11–15 mm) and peripheral arterial regions (*x*_p_, 2.41 ± 0.40; *r*_p_, 0.06 mm, range 0.01–0.10 mm). PA, pulmonary arterial tree; LPA and RPA, left and right pulmonary arterial trees, respectively; prox., proximal; peri., peripheral. *r*, vessel radius, presented as the mid‐point and its range for the same reason as described in the legend of Figure 1; *x*, radius exponent, defined by in equation (1 or 3; s and p, systemic and pulmonary, respectively.

*x *=**2.33 from equation 27 was compared with estimated and reported data of proximal systemic arterial *x*_s_, and proximal and peripheral pulmonary arterial *x*_p_, in Figure 4A (*r*_s_ > 1 mm) and Figure 4B, respectively. Equation 33 was plotted in Figure 4A together with reported and our estimated mean *x*_s_ after adjusting the range of *r*_s_ to the corresponding *Re* as follows. First in the proximal systemic artery, average *Re* values at the aortic bifurcation and in the common iliac arteries were reported to range between 400 and 1100 (mean, 730), and between 390 and 620, respectively (Nichols et al. 2011). Second, when *ρ* of 1.06 mg/cm^3^ and *μ* of 0.04 Poise (g cm^−3^ sec^−1^) are substituted for equation 32 (Horsfield and Woldenberg 1989; Kamiya and Takahashi 2007; Nichols et al. 2011), *Re* can be represented in the following equation:
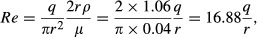
where *q* and *r* are given in cgs unit (Nichols et al. 2011). Mayrovitz and Roy (1983) reported the regression equation as *q*_s_ = 200 × *D*_s_^2.89^ in cgs unit, where *q*_s_ and *D*_s_ represented mean blood flow in an artery (cm^3^/sec) and its vessel diameter (cm) ranging between 0.0006 and 0.0108 cm, respectively, in an in vivo direct microscopic measurement of cremaster muscle arteries of rats. Applying the result reported by Mayrovitz and Roy to equation 35 by expanding the range of *r*_s_ to 0.1 mm, we were able to estimate *Re* as 4.2 (=16.88 × 200× (0.02)^2.89^/0.01) at *r*_s_ = 0.1 mm. Therefore, average *Re* is considered to reside within the range of 4 and 400 in the region of intermediate systemic arteries. Several coupled conditions of physiological Ω and *Re*s, under which equation 33 gives *x*_s_ of 2.7, are presented in Table 5. Thirdly, *Re* at *D*_s_ = 0.1 mm (*r*_s_ = 0.05 mm) in an intraorgan arterial bed was reported to be 0.1 by theoretical simulation by Kizilova (2006). Thus, average *Re* in arteries of *r*_s_ ≤ 0.1 mm is supposed to range approximately between 0.1 and 4. Values of *Re* in the range from 0.1 to 4 yielded *x*_s_ = 3.00 with equation 31 (Fig. 4A), where Ω_s_ spanned from 60 to 70 in peripheral systemic arteries (Table 4A).

**Table 5.** Coupled conditions of Ω and *Re* in the physiological mammalian systemic artery, which render *x* to get close to 2.70 as predicted by the rigid model.

Ω	5	20	40	60
*Re*	70	300	500	800
*x*	2.69	2.68	2.71	2.70

*Re*, Reynolds number, defined by equation 32; *x*, radius exponent, computed by equation 33 with coupled values of Ω and *Re* under *α *= 1.0. *α* and Ω represent exponent and proportional coefficients of the relationship between vessel length and radius, respectively, defined by equation 17.

## Discussion

Application of the least energy principle to *E* for both elastic and rigid arterial models yielded the optimal *x* for each model. Irrespective of whether it is elastic or rigid, these results in a single arterial model could successfully be extended even to an asymmetric fractal arterial tree system from the viewpoint of blood flow preservation at branchings. Neither blood temperature in equation 16 nor metabolic rates of *K*_b_ and/or *K*_w_ in equation 6 influenced optimal arterial fractal structures in this model analysis. We can regard *T* as independent of the radius in general. *K*_b_ and/or *K*_w_, whose estimated values based on experimental data were reported to be 4.34 × 10^3^ and 29.9 × 10^3^ erg cm^−3^ sec^−1^, respectively, by the detailed study of Mayrovitz and Roy (1983), were also independent of the radius in previous model studies (Murray 1926; Uylings 1977; Sherman 1981; Mayrovitz and Roy 1983; Griffith and Edwards 1990; Gafiychuk and Lubashevsky 2001). However, if the local environment happens to change 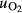 and *T* as well as *K*_b_ and *K*_w_, the optimal *x* cannot in all likelihood escape their influence. The Fåhræus‐Lindqvist effect (Dawson et al. 1999; Kamiya and Takahashi 2007; Nakamura et al. 2011; Nichols et al. 2011), which is well known as a non‐Newtonian effect, will yield a smaller *x* than the predicted value of 3.0 by equation 33 in the peripheral systemic arteries because this effect raises *Re* by significantly decreasing *μ* under the condition of 0.004 ≤ *r*_s_ ≤ 0.2 mm (Kamiya and Takahashi 2007; Nichols et al. 2011).

The arrangement of *x*_s_ obtained from two canine data sets (Sherman 1981; Milnor 1982) in Figure 3A proved similar to the inverse‐sigmoid curve in Figure 4A, suggesting a multifractal vascular system (Zamir 2001; Grasman et al. 2003). Equation 33 under several physiological conditions of Ω and *Re* (Table 5), which yields *x* around 2.7, partly explained quantitatively this tendency of reported or estimated *x*_s_ distribution at intermediate systemic arteries (0.1 < *r*_s_**≤ 1 mm) (Fig. 4A). Alternatively, we can get *Re* = 13.1 Ω by substituting both *x *=**2.7 and *α *= 1.0 into equation 33. Estimated mean *x*_s_ for proximal (*r*_s_ > 1 mm) and peripheral systemic arteries (0.004 ≤ *r*_s_**≤ 0.1 mm) from Milnor (1982) and Sherman (1981) agreed with the pertinent morphometric and hemodynamic data in the literature (Sherman 1981; Zamir and Brown 1982; Mayrovitz and Roy 1983; House and Lipowsky 1987; Kassab and Fung 1995; LaBarbera 1995; Nakamura et al. 2011), as indicated in Table 3A. While *x*_s_ for intermediate systemic arteries (0.1 < *r*_s_**≤ 1 mm) from Milnor's (1982) data was estimated to be a little smaller than the expected value of 2.7, that from the modified Mall's data (Sherman 1981) estimated as 2.2 turned out to be closer to that of more proximal elastic arteries (Table 3A). Because *x*_s_, which we think is largely affected by wall properties, would also vary among regions, animal species and, most of all, among individuals even with the same radius, the property of intermediate elastic‐muscular arteries might be identified not by the scale of *r*_s_ alone, but rather by *x*_s_ itself, as presented in Table 6. While *x*_s_ from the rank 4 (fifth generation) arteries of modified Mall's data was regarded as an outlier by the Q test in this analysis, Sherman originally mentioned that Mall's data of rank 3 (*r*_s_ = 0.096 mm) and four (*r*_s_ = 0.026 mm) arteries were questionable due to a systematic distortion in the size of some of the small arteries, since the dogs in Mall's study were killed by bleeding, with the result of probably pronounced vasoconstriction (Sherman 1981).

**Table 6.** Categorization of systemic arteries by radius exponent in the two canine data sets in literature.

Locality	References	*x*_s_	Range of radius (mm)	Expected wall property	Expected range of Reynolds number
Proximal		*x*_s_ ~ 2.3		Elastic	400–1100^1^
Dog	Milnor (1982)	2.32 ± 0.06	0.65–9.5		
Dog SMA	Sherman (1981)	2.30 ± 0.16	0.5–1.5		
Intermediate		*x*_s_ ~ 2.7		Elastic‐muscular	4^2^–400
Dog	Milnor (1982)	2.65	0.225		
Dog SMA	Sherman (1981)				
Peripheral		*x*_s_ ~ 3.0		Muscular (rigid)	0.1^3^–4
Dog	Milnor (1982)	2.95	0.075		
Dog SMA	Sherman (1981)	3.10 ± 0.24^4^	0.011–0.096		

*x*, radius exponent, defined by equation (1 or 3; s, systemic; SMA, superior mesenteric artery. Sources of data sets were table 2.3 (Milnor 1982) and table III (Sherman 1981).

^1^Nichols et al. (2011); ^2^estimated from equation 35 and Mayrovitz and Roy (1983); ^3^from Kizilova (2006); ^4^does not include the rank 4 arterial data (Sherman 1981) as an outlier.

Mean estimated *x*_p_ from the data of normal mammalian pulmonary arterial morphometry in the literature (Singhal et al. 1973; Horsfield 1978; Zhuang et al. 1983; Gan and Yen 1994; Jiang et al. 1994; Huang et al. 1996; Nakamura et al. 2011) focused around 2.3–2.5 in both proximal (*r*_p_ > 0.1 mm) and peripheral pulmonary arteries (0.01 ≤ *r*_p_ ≤ 0.1 mm) as displayed in Table 3B and Figure 4B. However, it will not be justified so easily to regard *x*_p_ as monofractal throughout the whole pulmonary arterial tree because the distribution of individually estimated *x*_p_ exhibited an actually large range at proximal pulmonary arteries, as presented in Figure 3B. Marked fluctuation of *x*_p_ at these proximal pulmonary arteries can be attributed to such factors as their varied elliptic cross section (Milnor 1982; Nichols et al. 2011), significant tapering structure of the main pulmonary artery toward its first branches (Milnor 1982; Dawson et al. 1999), and the inherent constraints imposed, more or less, on these vessels by the shape of the thoracic cage, lungs, and heart (Singhal et al. 1973). However, estimated peripheral *x*_p_ of three humans (Singhal et al. 1973; Horsfield 1978; Huang et al. 1996) converged at 2.41 ± 0.18 in the range of *r*_p_ below 0.1 mm, as shown in Figure 3B, where *x*_p_ of a dog (Gan and Yen 1994) and a cat (Zhuang et al. 1983) also stayed at the same level in the similar range of *r*_p_. Stable and consistent peripheral *x*_p_ among normal humans and these mammals are probably due to similar respiratory physiology and pulmonary circulation (Table 3B).

Our estimates of *α* and Ω agreed with the results reported by previous studies (Suwa and Takahashi 1971; Dawson et al. 1999), as indicated in Table 4A and B. We could confirm the empirically reported relationship between *r* and *l* as written in equation 17 by large correlation coefficients (*r*) between ln*r* versus ln*l* (Table 4A and B). Although several estimated values of Ω differed to a considerable degree between proximal and peripheral pulmonary arteries, the difference was partly due to the much smaller number of peripheral samples in the data than proximal samples. As indicated in Tables 2 and 4, *α*_p_ actually stayed slightly below 1.0 or ~0.8, which provides *x*_p_ ≈ 2.27 (Fig. 4B) from equation 27. The range of *α* from 0.72 to 1.23, as indicated in Tables 2 and 4, yields *x* in the elastic arterial model from 2.24 to 2.41. On the other hand, the same range of *α* from 0.72 to 1.23 causes only a trivial deviation of *x* in the rigid model (eq. 33; for example, *x* resulted in 2.348–2.350 (Ω = 1.5; *Re* = 1000) and 2.996–2.997 (Ω = 50; *Re* = 4).

Our elastic arterial model explains *x *=**2.33 by Bernoulli's effect. However, it has conventionally been explained by Uylings’ theoretical prediction under the condition of *complete turbulence* in a rigid cylindrical vessel (Uylings 1977), one of the most influential theories (Sherman 1981; Horsfield and Woldenberg 1989; Huang et al. 1996; Bennett et al. 2000; Olufsen et al. 2000). But it still remains controversial to explain the radius exponent reported in proximal systemic arteries as well as in the whole pulmonary arterial tree by the presence of turbulence alone (Caro et al. 1978; Roy and Woldenberg 1982; Horsfield and Woldenberg 1989; Nichols et al. 2011). Because the peak *Re* in human systemic and pulmonary arteries is estimated as <2000 except for the region just above the aortic valve (Caro et al. 1978; Nichols et al. 2011), it conflicts with the presence of continuous turbulence. Horsfield and Woldenberg (1989) stated that turbulence by itself could not fully account for the coexistence of both *x*_p_ = 2.3 ± 0.1 and *Re* < 2000 at the same time in the case of pulmonary arteries, while several more direct effects resulting from arterial wall elasticity should also intervene.

The result in our rigid arterial model led us to represent *x* with a novel function of *Re* and Ω, as presented in equation 33. This equation provides the general solution of optimal *x* under various blood flow levels, involving both Murray's and Uylings’ theories. First, Murray's ∂*C*/∂*r *=**0 in equation 12 is a particular solution of ∂*E*/∂*r *=**0 of equations (11 and 21. The ratio of *U*_K_ over Δ*U*_P_ in equation 21 is proportional to *Re* when *α* is equal to 1.0:
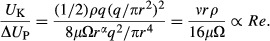


Because the result of ∂*E*/∂*r *=**0 in equation 21 approximates that of ∂*C*/∂*r *=**0 in equation 7 under the condition of *U*_K_ ≪ Δ*U*_P_ and *Re* becomes sufficiently low as shown in equation 36, *x* reaches 3.00, which Murray's (1926) law eventually advocates. Moreover, Uylings’ (1977) theory is another particular solution of ∂*E*/∂*r *=**0 under *Re* → ∞. *E* in equation 21 appears similar to equation 20 under the inverse condition of Δ*U*_P_ ≪ *U*_K_, where *Re* rises sufficiently high as in equation 36, and *x* approaches 2.33, which is compatible with what Uylings’ model relates. The complete turbulence in equation 21 means that blood flow closely mimics the ideal fluid with Bernoulli's principle.

However, our model does not explain the actual morphometric data of *x *<**2 or > 3 observed in some particular states of normal mammalian vasculatures (Woldenberg 1983; LaBarbera 1995; Dawson et al. 1999; Bennett et al. 2000). Furthermore, our theoretical results do not necessarily account for radius exponents found in some pathophysiological arterial remodeling processes of human and/or mammalian diseases. Ghorishi et al. (2007) reported the mean *x*_p_ to be 1.671, which is not accountable by our model, at 0.01 ≤ *r*_p_ ≤ 10 mm in cast morphometry of a 2‐month‐old lamb with secondary pulmonary hypertension (PH) caused by surgically produced left‐to‐right (L–R) shunt in the fetal period. However, the same shunt lamb's *x*_p_ targeted 3.0 at the most peripheral pulmonary arterioles (*r*_p_ ≤ 0.01 mm) in their Figure 2B (Ghorishi et al. 2007), while its *x*_p_ stayed around 2.0–2.3 at *r*_p_ from 0.4 to 3 mm. Our recent analysis of hemodynamic data revealed that the estimated *x*_p_ in patients with congenital L–R shunt defects increased gradually from 2.5 to 3.0 in peripheral pulmonary resistive arteries whose radius range was assumed to be 0.01 ≤ *r*_p_ ≤ 0.1 mm in accordance with the severity of secondary PH, while *x*_p_ of controls remained at 2.2 (Nakamura et al. 2011). Congenital L–R shunt defects induce medial hypertrophy and vasoconstriction of intra‐acinar arteries (0.004 ≤ *r*_p_≤ 0.1 mm) with secondary PH a few months after birth along the postnatal course of the disease (Michel et al. 1985; Ghorishi et al. 2007). Both of these pathophysiological changes are considered to induce strikingly decreased elasticity and increased rigidity of intra‐acinar arterial wall properties.

There are limitations for this model analysis. The energy function *E* in this analysis did not take into account non‐Newtonian effects and energy dissipations due to either turbulence or arterial ramifications. Bernoulli's equation is not necessarily guaranteed through arterial ramifications but holds true for a single elastic artery and/or valve (Lima et al. 1983a,b; Bermejo et al. 2002). Because most morphometric studies were performed with formalin‐fixed specimens or by the resin cast method, their data do not necessarily reflect actual in vivo pulsatile hemodynamic realities (Suwa and Takahashi 1971; Singhal et al. 1973; Bennett et al. 2000; Nichols et al. 2011). The concept of the optimality principle itself cannot escape some limitations. First, the in vivo vascular system must meet multiple functional requirements that are not fully expressible in terms of mathematical optimality conditions. Second, there is no guarantee that the vascular system will attain a mathematically deduced optimal state: in other words, the actual vessel structure of a living body shows significant asymmetry and heterogeneity (Woldenberg 1983; Griffith and Edwards 1990; Dawson et al. 1999; Bennett et al. 2000).

Combining a novel modification of Murray's law and a scaling exponent between vessel radius and length, our model proposes a new approach to account for the topically different hemodynamic conditions in elastic and rigid arterial models leading to the radial exponent 2.33 and continuously changing from 2.33 to 3.0 as a function of Reynolds number, respectively. They are known as Uylings’ law for turbulence and Murray's law for laminar flow, respectively. In spite of the above‐mentioned limitations, our model study explains the radius exponent in the elastic arterial system not by turbulence but by its elasticity and Bernoulli's effect, and our novel expression of *x* in rigid arteries is a step for further understanding the relationship among the fractal dimension, the situation of blood flow, and the arterial scaling structure. From the practical viewpoint, the results of theoretical approaches including ours will enable accurate estimation of blood velocity and its feasible distribution, and could be applied to pharmacodynamics in the systemic and pulmonary circulation. Equations (27 and 29 would offer a surgically preferable reconstructive design in terms of proximal arterial branching structures, and help alleviate the total cardiac work imposed and necessary to maintain sufficient blood circulation. Reflecting both the elastic and rigid arterial models in the industrial design of the artificial vascular structure would also contribute to making prostheses hemodynamically more efficient. We hope that this model analysis will eventually contribute on a theoretical basis to further biological and medical knowledge.

## Acknowledgments

The corresponding author (Y. N.) thanks S. Naritaka, Y. Yogo, and Y. C. Nakamura for their continued encouragement. The authors are indebted to the anonymous reviewers of this article for his (or her) deep interest and inspiring comments and advice. This helped us to improve and finalize the content and make findings, which otherwise might have escaped our observation.

## Conflict of Interest

None declared.

## Appendix

Under the condition of *α* = 1.0 at the exponent in equations 17,30 can be transformed to

Using a real number *ε* as a proportional coefficient, equation 1 can be written as

When *f* is defined as

as a method to calculate equation (A1, the equation is simplified as

To make the succeeding composite functions easier or clear‐cut for differential calculus, we tentatively define *g*,* y*, and *z* as follows for convenience:

Equations (A2, A3, and A6 give us

Equations (A4 and A7 lead eventually to

Eliminating *r* in equation (A9 by equation (A10 yields

Partial differentiation of *g* in equation (A11 with respect to *y* and equation (A8 results in

On the other hand, equations (A6, A7, and A8 provide

With equations (A5, A12, and A13) we can describe *x* as
